# An Examination on the Transmission of COVID-19 and the Effect of Response Strategies: A Comparative Analysis

**DOI:** 10.3390/ijerph17165687

**Published:** 2020-08-06

**Authors:** Yi-Tui Chen, Yung-Feng Yen, Shih-Heng Yu, Emily Chia-Yu Su

**Affiliations:** 1 Department of Health Care Management, College of Health Technology, National Taipei University of Nursing and Health Sciences, Taipei 10845, Taiwan; yitui@ntunhs.edu.tw (Y.-T.C.); dam37@tpech.gov.tw (Y.-F.Y.); 2 Section of Infectious Diseases, Taipei City Hospital, Yangming Branch, Taipei 11146, Taiwan; 3 Institute of Public Health, National Yang-Ming University, Taipei 11221, Taiwan; 4 Department of Business Management, National United University, Miaoli 36003, Taiwan; 5 Graduate Institute of Biomedical Informatics, College of Medical Science and Technology, Taipei Medical University, Taipei 11031, Taiwan; 6 Clinical Big Data Research Center, Taipei Medical University Hospital, Taipei 11031, Taiwan

**Keywords:** COVID-19, transmission, mitigation effectiveness, pandemic severity, viral testing

## Abstract

The major purpose of this paper was to examine the transmission of COVID-19 and the associated factors that affect the transmission. A qualitative analysis was conducted by comparing the COVID-19 transmission of six countries: China, Korea, Japan, Italy, the USA, and Brazil. This paper attempted to examine the mitigation effectiveness for the transmission of COVID-19 and the pandemic severity. Time to reach the peak of daily new confirmed cases and the maximum drop rate were used to measure the mitigation effectiveness, while the proportion of confirmed cases to population and the mortality rate were employed to evaluate the pandemic severity. Based on the mitigation effectiveness, the pandemic severity, and the mortality rate, the six sample countries were categorized into four types: high mitigation effectiveness vs. low pandemic severity, middle mitigation effectiveness vs. low pandemic severity, high mitigation effectiveness vs. high pandemic severity, and low mitigation effectiveness vs. high pandemic severity. The results found that Korea and China had relatively higher mitigation effectiveness and lower pandemic severity, while the USA and Brazil had the opposite. This paper suggests that viral testing together with contacts tracing, strict implementation of lockdown, and public cooperation play important roles in achieving a reduction in COVID-19 transmission.

## 1. Introduction

Since the first confirmed case occurred in China, more than 170 days have passed. The disease has rapidly diffused into almost every corner in the world. As of 10 June 2020, 6,931,000 confirmed cases and 400,857 deaths in 216 countries/areas/territories due to COVID-19 were reported [1]. The pandemic seems to be continuing with no sign of stopping as the number of new cases around the world was still increasing as of June 2020. Some countries have recovered from the pandemic, such as China, Japan, and Korea, as their new confirmed cases have gradually approached zero. The number of daily new cases in China was in single digits and in the tens in Japan and Korea at the beginning of June 2020. In contrast, as of 10 June 2020 the pandemic was still ongoing in some countries like Brazil and USA.

At the onset of a pandemic, the prompt and correct response strategy may be very important to effectively slow down the transmission of COVID-19. Identifying the correct response strategies from the previous cases may be of significance to help post-infected countries strengthen pandemic prevention. Liu [2] presented a model to analyze the factors affecting the transmission of COVID-19 among cities. Chimmula and Zhang [3] predicted the transmission of COVID-19 in Canada by using the long short-term memory (LSTM) networks, a deep learning approach. Their results suggested that the pandemic of COVID-19 in Canada would end around June 2020. Lotfia et al. [4] presented a review discussing the transmission routes of COVID-19. Shim et al. [5] investigated the growth rate of the outbreak and estimated the reproduction number by using the daily confirmed cases of COVID-19 in Korea.

However, very few studies have focused on mitigation effectiveness and the pandemic severity in responding to the COVID-19 attack. Various response strategies may yield different effectiveness in mitigating COVID-19 transmission, including lockdown, stay-at-home, contact tracing, school and workplace closures, social distancing, limitation on flights, etc. [1,6]. Moreover, the variation of the response strategies also may bring about different levels of impact on the pandemic severity. Thus, the major purpose of this paper was to examine mitigation effectiveness and the pandemic severity in association with different mitigation strategies by using six countries for case studies. This paper employed the time to reach the peak of daily new confirmed cases and the maximum drop rate to measure the mitigation effectiveness and the proportion of confirmed cases to population and the mortality rate to evaluate the pandemic severity.

Briefly, the purpose included: (1) To examine the effectiveness of the mitigation of the transmission at the early stage, the latter stages, and throughout the whole period in the countries selected; (2) To investigate the pandemic severity at the early stage, the latter stage, and throughout the whole period in the countries selected; (3) To examine the association of the pandemic severity at the early stage with the pandemic severity at the latter stage; (4) Based on the effectiveness and the pandemic severity, a discussion is conducted, integrating the response strategies adopted in each country. This paper employed a qualitative analysis to compare the pandemics of six countries: China, Korea, Japan, Italy, USA, and Brazil. The results derived from the analysis in this paper may be used as a reference to prevent the spread of the virus in the other countries.

## 2. Research Methods

### 2.1. Criterion of Sample Selection

Up to 7 June 2020, more than 200 countries/areas/territories have reported confirmed cases of COVID-19. In this paper, we selected six countries for case studies to analyze the impact of the response strategies on the transmission of COVID-19: China, Korea, Japan, Italy, the USA, and Brazil. The criterion for selecting these countries was based on the early outbreak of COVID-19 or the severity of the attack.

According to the World Health Organization (WHO) [1], the world’s first case of the coronavirus disease was detected and confirmed in China in the end of 2019. By 7 June 2020, the total confirmed cases in China reached to 84,629 cases, ranked the top in the Western Pacific, ahead of Japan and Korea.

Japan and Korea were selected due to their earlier confirmed cases of COVID-19 infection compared with those in other countries. They confirmed their first cases of COVID-19 almost simultaneously, but the rise in the rates of new cases in the early stages and the decline in the rates of new cases at the latter stages between the two countries differed greatly. The disease of COVID-19 diffused rapidly in Korea by the end of February, 2020 but in Japan it started to spread on a large scale only after the end of March.

Italy’s outbreak began in March 2020, and in a short time, daily new cases of COVID-19 increased very fast. It increased sharply from 466 cases on 4 March to the peak of 6557 cases on 22 March 2020. On 28 March 2020, the total confirmed case reached to 86,498 in Italy, exceeding the confirmed cases of 82,213 in China and leading the world in cases that day. On the next day (29 March 2020), Italy was replaced by the USA as the country with the most cases. On 29 March 2020, the total confirmed cases in the USA reached 103,321, with 18,093 new cases causing an increase from the 85,228 cases reported on 28 March 2020 [1]. Since then, the USA has been the leader in total confirmed cases of COVID-19. As of 7 June 2020, the USA still ranked at the top for total confirmed cases at 1,886,794 and total deaths of 109,038, followed by Brazil that had total confirmed cases of 645,771 and total deaths of 35,026.

### 2.2. Data Collection

The data for the number of new confirmed cases and total confirmed cases on each day were extracted from the daily situation reports of the World Health Organization [1]. The number of observation points is the same across the six countries, covering 133 days for each country to have the same comparison scale. The dates of the first confirmed cases were identified for each country and are listed in Table 1.

The outbreak of COVID-19 was initiated from Hubei Province, China in December 2019 [1]. The information released from the WHO demonstrates that 44 cases of pneumonia of unknown etiology were detected in Wuhan City, Hubei Province of China on 31 December 2019. On 7 January 2020, a new type of coronavirus was isolated and identified. The statement, “Symptom onset of 41 confirmed cases ranges from 8 December 2019 to 2 January 2020” was released by the WHO [1] on 11 January 2020. Thus, the first case of the disease is estimated to have taken place on 8 December 2019 in China.

In a short time, the COVID-19 spread to many other countries. On 15 January 2020, the Japanese Ministry of Health, Labor and Welfare reported an imported confirmed case of a novel coronavirus (2019-nCoV) that was detected outside of China. This implies that Japan’s outbreak started on 15 January 2020. Following confirmation of COVID-19 disease in Japan, Korea informed the WHO of a confirmed case of a novel coronavirus (COVID-19) on 20 January 2020.

According to the data of the Situation reports released by the WHO, the first confirmed case in the USA was reported on 23 January, 31 January for Italy, and 27 February 2020 for Brazil. The data were extracted from the starting dates of the pandemic for each country to the last observation point of 7 June 2020.

### 2.3. Indicators for Mitigation Effectiveness of COVID-19 and Pandemic Severity

This paper employed a 3-day moving average calculated by averaging the number of that day, the day before and the next day to understand the trend of daily new COVID-19 cases. This approach may prevent reporting errors or testing errors.

In general, the response strategies adopted by each country are enforced step by step from moderate to stringent until the date when new confirmed cases decreased. Most countries in general tried various strategies and waited for the outcome, and thus response strategies changed over time before the peak of the pandemic and became stable after the peak. Time spent to reach the peak and to become stable may reflect the effectiveness in mitigating the transmission of the infection. However, new confirmed cases in some countries still remained high on the date of the final observation point, and time spent to reach a stable state of the pandemic after the peak became unmeasurable. Thus, the pandemic was separated into two stages: the early stage from the first confirmed case to the date when the peak of the daily new cases takes place, and the latter stage starting from date of the peak to the 133rd day since the first confirmed case.

The mitigation effectiveness at the early stage $E_{e}$ is measured by time spent to reach the peak and mitigation effectiveness at the latter stage $E_{f}$ is measured by the maximum drop rate during the peak and the final observation point (7 June 2020). A scatter diagram is depicted based on $E_{e}$ and $E_{f}$ to determine the overall mitigation effectiveness $E_{o}$ where the subscripts *e*, *f*, and *o* denote the early stage, the latter stage, and the whole period.

Pandemic severity $S_{i}$ is measured by the proportion of total confirmed cases *N* to population *P* each day, expressed as

(1) $$S_{i}=\frac{N_{i}}{Pt_{i}}$$

where *t* denotes days covered by the early stage (*i = e)*, the latter stage (*i = f)*, or the whole period (*i = o*).

The mortality rate *d* of the whole period is also employed to evaluate pandemic severity, calculated by the proportion of total deaths *D* to population *P* each day, expressed as

(2) $$d=\frac{D}{Pt}$$

where *t* represents days covering the whole period.

## 3. Results

**Figure 1:**
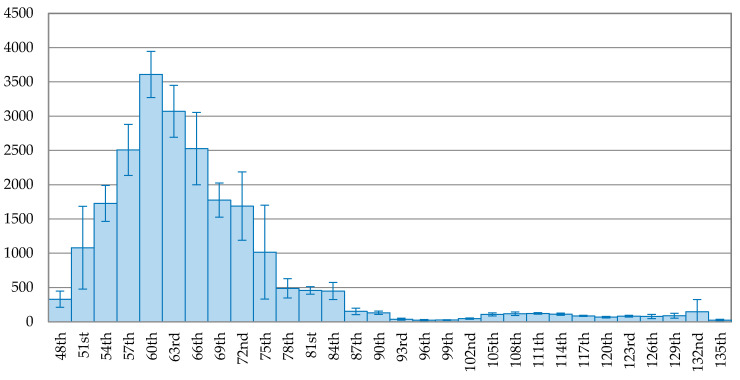
The trend of the 3-day average of new confirmed cases with error bars in China.

**Figure 2:**
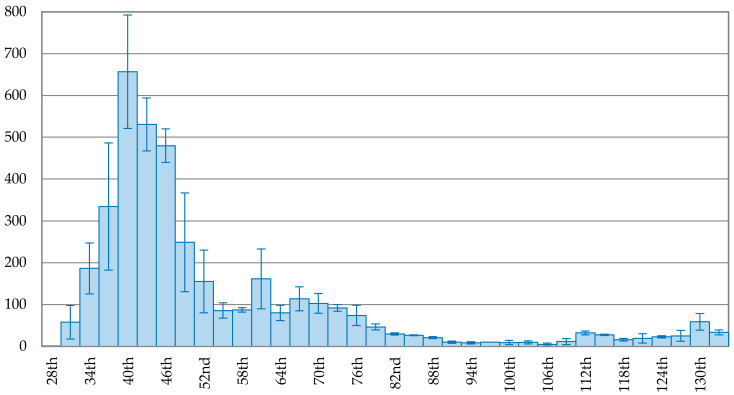
The trend of the 3-day average of new confirmed cases with error bars in Korea.

**Figure 3:**
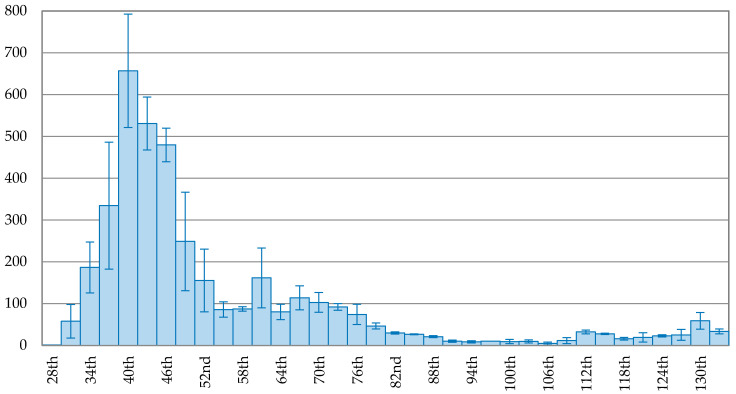
The trend of the 3-day average of new confirmed cases with error bars in Japan.

**Figure 4:**
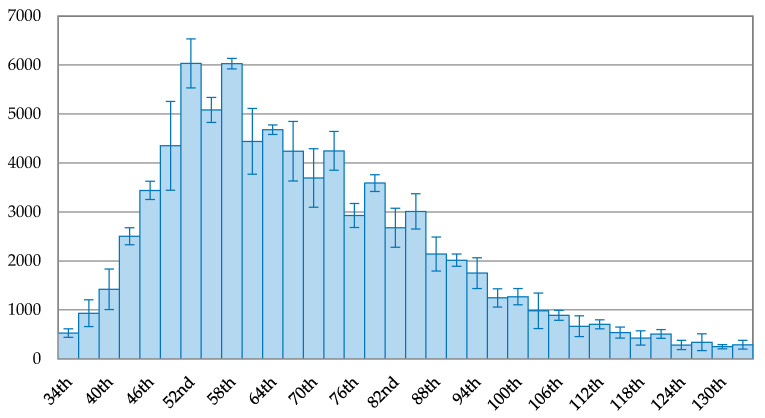
The trend of the 3-day average of new confirmed cases with error bars in Italy.

**Figure 5:**
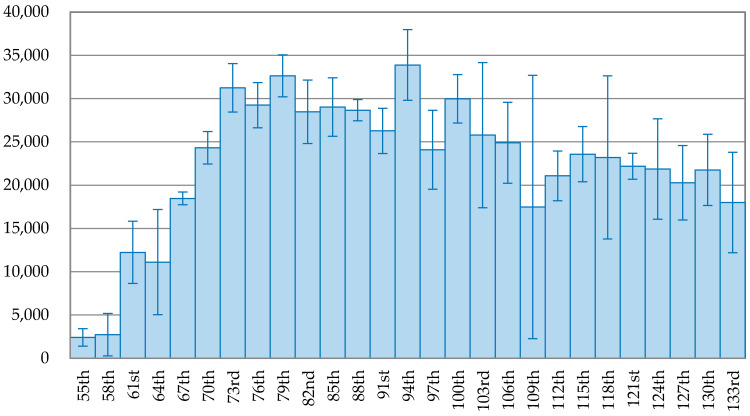
The trend of the 3-day average of new confirmed cases with error bars in the USA.

**Figure 6:**
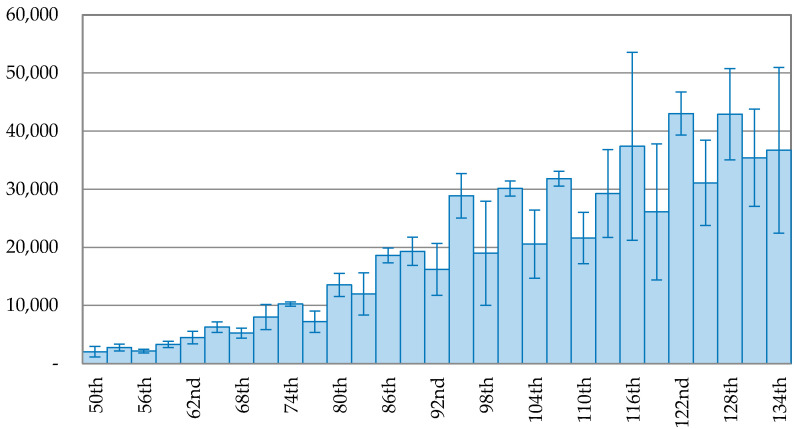
The trend of the 3-day average of new confirmed cases with error bars in Brazil.

The trend of the 3-day average of new confirmed cases is depicted on Figure 1, Figure 2, Figure 3, Figure 4, Figure 5 and Figure 6 for each country. The variable on the *X*-axis represents the days since the first confirmed case and the *Y*-axis is the number of the 3-day average of new confirmed cases. A brief comparison among the six countries demonstrates that the daily new cases in China, Japan, Korea, and Italy started to decline but the daily new cases did not start to decline for USA and Brazil. Figure 1 reveals that the pandemic in China is successfully under control, as the curve in the latter stage keeps going downward. The peak of daily new confirmed cases occurred on the 60th day (5 February 2020) and after that, the new confirmed cases continuous reduced. The daily new confirmed cases dropped to below 1% of the peak on the 96th day (12 March 2020), 36 days since the peak.

The trend of new confirmed cases in Korea is depicted in Figure 2, showing a continuous decreasing trend after the peak. On the 102nd day (1 May 2020), the 3-day average of new confirmed cases dropped to 6, about 0.95% of the peak (657 cases). Compared to Korea, Japan took more time to reach the peak, indicated in Figure 3. The trend of new confirmed cases in Japan fluctuated after the peak. On the 129th day (24 May 2020), the 3-day average of new confirmed cases dropped to 23 cases, about 3.56% of the peak, reaching a maximum drop rate.

Figure 4 demonstrates that Italy had two peaks of the 3-day average of new confirmed cases, occurring on the 52nd day (22 March 3030) with 6034 cases and the 56th day (28 March 2022) with 6029 cases. Thus, the data for the 52nd day was used as the peak for comparison in this paper. The overall view on the trend of new confirmed cases in Italy shows a continuous downward trend. On the 115th day (24 May 2020), the drop rate reached 91.08% of the peak with 538 new confirmed cases. The maximum drop rate after the peak in Italy was 95.87%, occurring on the 130th day (8 June 2020).

Figure 5 and Figure 6 depict the trend of the 3-day average of new confirmed cases in the USA and Brazil. The peak of new confirmed case in the USA was 33,882 cases, occurring on the 94th day (26 April 2020). The trend fluctuated after the peak with a low declining rate. On the 109th day (11 May 2020), the number of new confirmed cases was 17,471, accounting for 51.56% of the peak, falling at the bottom of the trend after the peak. However, it immediately rose up again for six days and then fluctuated. On the 136th day (7 June 2020), the number of newly infected people increased to 25,970 cases, accounting for 76.65% of the peak. Figure 6 shows that the outbreak of COVID-19 in Brazil kept growing and reached the peak of 45,680 new confirmed cases on the 129th day (4 July 2020). After that, the new confirmed cases dropped to 26,051 cases on the 132nd day, but rose again on the 133rd day (8 July 2020).

Mitigation effectiveness at early stage $E_{e}$ and mitigation effectiveness at latter stage $E_{f}$ are summarized in Table 2. They demonstrate that Korea had the best mitigation effectiveness by spending the shortest time of 40 days to reach the peak of new confirmed cases, while the peak had not yet occurred in Brazil. China, Japan, Italy, and the USA followed Korea, spending 60, 87, 52, and 97 days to reach the peak, respectively. As to Brazil, the number of new confirmed cases kept growing and reached a climax on the 129th day (4 July 2020). Brazil took the longest time to reach the peak and thus ranked at the bottom for mitigation effectiveness at the early stage $E_{e}$.

Mitigation effectiveness at the latter stage $E_{f}$ measured by the maximum drop rate for the USA was only 48.44%, it occurred on the 109th day (11 May 2020). Korea has the best $E_{f}$ (maximum drop rate 99.55%), ahead of China (99.37%), Japan (96.44%), Italy (95.87%), the USA (48.44%), and Brazil (38.55%).

Table 2 also demonstrates (1) the time until a drop of 50% from the peak, (2) the time until a drop of 80% from the peak, (3) the time until a drop of 90% from the peak, and (4) the time until a drop of 95% from the peak to analyze the relative mitigation effectiveness at the latter stage. China and Korea took 9 days to reduce the transmission from the peak by 50%, Japan 15 days, and Italy 24 days. However, USA and Brazil had not yet attained a reduction of the pandemic by 50% on the final observation of the 133rd day since the first confirmed case.

The further drop of daily new confirmed cases from the peak by 80% took China, Korea, Japan, and Italy 16, 13, 25, and 51 days and the drop of the pandemic by 90% took China, Korea, Japan, and Italy 27, 39, 36, and 63 days, respectively. China took 27 days to reduce the new confirmed cases by 95% after the peak, and the same drop took 42 days for Korea and Japan while Italy took 72 days.

**Figure 7:**
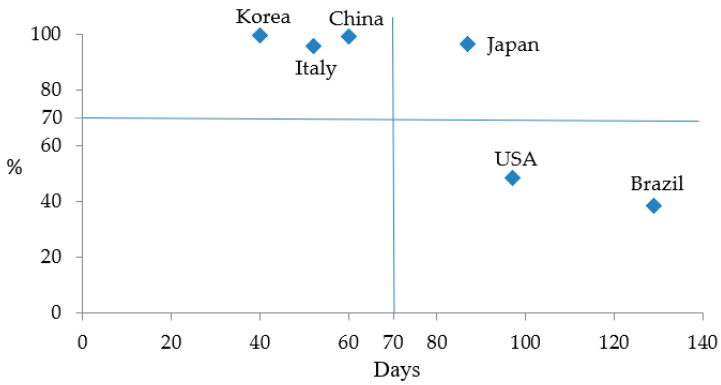
The mitigation effectiveness for the transmission of COVID-19.

The overall mitigation effectiveness $E_{o}$ was obtained by a scatter diagram. Figure 7 shows mitigation effectiveness at the early stage $E_{e}$ (unit: days) on the horizontal axis against mitigation effectiveness at the latter stage $E_{f}$ on the vertical axis. The middle value of 71 days of $E_{e}$ and 70% of $E_{f}$ were used as separation lines to classify the countries into high, middle, and low overall effectiveness $E_{o}$. In Figure 7 China, Korea, and Italy are categorized into high $E_{o}$, Japan middle $E_{o}$ due to low effectiveness at the early stage $E_{e}$, and the USA and Brazil are classified into low $E_{o}$ due to low mitigation effectiveness at both early and latter stages. The result of $E_{o}$ is listed in Table 2.

The pandemic severity at the early stage $S_{e}$, the latter stage $S_{f}$, and the whole period $S_{o}$ were calculated according to Equation (1) and the results are listed in Table 3. Among these countries, China ranked the lowest $S_{e},\;S_{f}$, and $S_{o}$, amounting to 0.28, 0.57, and 0.32 cases per million persons per day, ahead of Japan, Korea, and the other countries. In contrast, Brazil had the highest $S_{e}$ of 54.59, $S_{f}$ of 148.69, and $S_{o}$ of 74.87 cases per million persons per day. This paper also employed the mortality rate *d* to measure pandemic severity, and the results are also listed in Table 3. By the date of the 133rd day since the first confirmed case for each country, the mortality rates in China, Korea, and Japan were 0.02, 0.04, and 0.05 cases per million persons per day, much lower than those of the other three countries. In Italy, 4.24 persons died of COVID-19 per million per day before the 133rd day since the first confirmed case, the highest in the six countries. Furthermore, the much lower value of pandemic severity $S_{o}$ and the mortality rate *d* by the date of the 133rd day in China, Japan, and Korea implies that China, Japan, and Korea have recovered from the pandemic. As the values of the pandemic severity and mortality rates in China, Korea, and Japan are not of the same magnitude as those in Italy, the USAl and Brazil, this paper categorized the last three countries as high pandemic severity and the first three as low pandemic severity.

To have a clear understanding of the impact of the pandemic severity at the early stage on the latter stage, a correlation test of the pandemic severity between the two stages was conducted. The results found that the Pearson coefficient was 0.9979 and the coefficient of determination was 0.9959, implying the pandemic severity at the early stage may positively affect the pandemic severity at the latter stage. The results of Zhang et al. [7] by using the COVID-19 data from 436 cities in China found that the early growth in the first 100 confirmed cases may predict the pandemic size at the subsequent stages. The high correlation of pandemic severity between the early stage and the latter stage suggests that the response strategy should be urgently implemented as early as possible to prevent the expansion of the transmission of the infection.

## 4. Discussion

Based on the overall mitigation effectiveness $E_{o}$ and the overall pandemic severity $S_{o}$ of the COVID-19 transmission, the six countries are categorized into four types as shown in Table 4: Type I, high mitigation effectiveness and low pandemic severity (China and Korea); Type II, middle mitigation effectiveness but low pandemic severity (Japan); Type III, high mitigation effectiveness but high pandemic severity (Italy); and type IV, low mitigation effectiveness and high pandemic severity (the USA and Brazil).

### 4.1. Type I: High Mitigation Effectiveness and Low Pandemic Severity

The high mitigation effectiveness $E_{o}$ for the COVID-19 transmission and low pandemic severity $S_{o}$ in China and Korea may be attributed to the high viral testing rates and effective contacts tracing implemented. In addition to viral testing performed to identify the infected, the other non-pharmaceutical interventions such as lock down, social distancing, and school closure also play an important role in reducing the virus transmission.

Korea’s first confirmed case was identified on 20 January 2020 and reported to the WHO on 21 January 2020. At the beginning of the outbreak, only those who had symptoms and contact with confirmed cases were required to perform viral tests. After 12 February 2020, testing policies were revised and testing became open to the general public [6]. The tested cases increased from 21 on 23 January 2020 to 9772 on 18 February 2020, and then exponentially increased to 94,055 on 29 February 2020 when the peak of the new confirmed cases took place. After the peak, the COVID-19 tests kept growing exponentially and reached 1,012,769 cases on 7 June 2020 [8]. In addition, the response strategy adopted by the Korean government included raising the COVID-19 alert level to the highest (Level 4), the implementation of social distancing measures, restricting public transportation, cancellation of social events, and temporary closure of school activities, which were helpful in improving mitigation effectiveness and reducing pandemic severity. Shim et al. [5] suggested that the implementation of social distancing measures and other non-pharmaceutical interventions in Korea may help control the outbreak of the pandemic.

China took longer than did Korea to reach the peak, but took a shorter time to reduce the new confirmed cases by 90%. Their overall pandemic severity was also the lowest among the six countries. When the first confirmed case of COVID-19 took place in China in the end of 2019, the cause was unknown and not clear. It was novel to both China and the world and thus it was difficult for China to take appropriate response strategies at the early stage of the pandemic. Thus, it took China 60 days to reach the peak of new confirmed cases.

However, China immediately closed the concerned market on 1 January 2020 for environmental sanitation and further hygiene investigations were performed after detecting the cases of pneumonia of unknown etiology in Wuhan City. More than 35 infrared thermometers were installed in associated traffic systems including airports, railway stations, bus stations, and ferry terminals. Health education was promoted for the prevention of COVID-19 infection [1]. On 23 January 2020, the Chinese government announced a “metropolitan-wide quarantine” of the city of Wuhan that regulated all public transportation in the city and intercity links to be terminated [9]. Strict control to avoid frequent interpersonal contacts was implemented nationwide [10,11,12]. Accompanied with massive viral tests performed, China used 1800 teams of five epidemiologists each to trace contacts with confirmed cases daily [13].

At the beginning of February 2020, Hubei province accounted for more than 60% of the total new confirmed cases and each day thousands of new cases were reported [1]. Through the implementation of the strict control over the transmission of COVID-19, the daily new cases declined sharply, and the Wuhan lockdown was officially ended on 8 April 2020. At the end of May 2020, Wuhan city had no new cases to report [14].

Based on the pandemic severity in Korea and China, this paper suggests that Korea and China are basically largely recovered from the pandemic. This conclusion is the same as those of Kupferschmidt and Cohen [15] and Zhang et al. [7]. The success of the fight against COVID-19 in China and Korea may be due to high numbers of viral tests together with an effective tracing system as well as other mitigation strategies such as the lock down of cities, wearing masks, and social distancing to prevent the transmission of infection.

### 4.2. Type II: Middle Mitigation Effectiveness but Low Pandemic Severity

The analysis in this paper finds that Japan had a middle $E_{o}$ for the mitigation of COVID-19 transmission, but the $S_{o}$ in Japan was only 0.95 cases per million persons per day, putting then in second place. A middle $E_{o}$ was caused by the longer time to reach the peak (97 days) in Japan. The longer time to reach the peak for Japan may be attributed to the low test rate at the early stage of the outbreak. The government guidelines stipulated that only people having a fever of at least 37.5 °C for four consecutive days were allowed to consult a public health center for testing [16]. The viral test for COVID-19 was zero before 3 March 2020 based on the data released by Hasell et al. [17]. During the period of 4 March–1 April 2020, the test ratio was less than 0.01 per thousand people. Based on the data provided by Ministry of Health, Labor and Welfare [18], total number of tested cases reached 369,190 during the period of 2/18–5/16. At the beginning of the infection, the test capacity in Japan was less than 5000 cases per day and the actual tested cases ranged from 505–2540 cases per day before 27 March. After 28 March 2020, the tested cases increased sharply to a maximum of 9432 on 22 April 2020. Considering the lower number of new confirmed cases in Japan compared to that in Korea before March 2020, this paper suggests that the number of new confirmed cases may be under-estimated due to insufficient test capacity in Japan. Thus, many infected people were hidden and became a source of infection. As a consequence, more and more people were infected later and delayed the peak.

In the beginning of March 2020, the Japanese government attempted to continue planning for the Olympic Games based on the original schedule and declined to declare a state of emergency. Thus, no policy involving lockdown or staying at home was instituted. On 24 March 2020, the Olympic games extension was announced. After that, the tests for COVID-19 increased substanstially. The test ratio increased from 0.01 on 1 April 2020 to 0.04 per thousand people on 20 April 2020 [17]. On 7 April 2020, Japan announced a state of emergency and upgraded it to a nationwide measure on 16 April, and extended it till 31 May 2020 on 4 May 2020. The reduction in new confirmed cases at the end of April and May 2020 was obvious. Based on the pandemic trend in Japan, this paper suggests that the delay in implementing response strategies in Japan was the second cause for the delay of the peak.

### 4.3. Type III: High Mitigation Effectiveness but High Pandemic Severity

The mitigation effectiveness $E_{o}$ for the COVID-19 transmission in Italy was high, but the pandemic severity $S_{o}$ was also high. The time to reach the peak and to decline were acceptable compared to other countries. The maximum drop rate for Italy was 88.95%, lower than that of China, Korea, and Japan, but much higher than that of the USA and Brazil. The pandemic severity $S_{o}$ in Italy ranked second, amounting to 30.10 cases per million persons per day. This implies that many more people were infected every day compared to other countries, especially China, Korea, and Japan.

The first confirmed case of COVID-19 in Italy was reported to the WHO on 31 January 2020. At the onset of the COVID-19 outbreak, the Italian Government presented some emergency response strategies including school closure and discouraging public activities to prevent the transmission of the infection [19].

On 3 March 2020, eleven towns in North Italy announced quarantine to control the rapid transmission of COVID-19 [20]. However, many Italian citizens neglected the request of quarantine and continued their daily routine activities without change. Six days later (9 March 2020), the national quarantine was announced in Italy and the lockdown of cities was imposed in association with the restriction and separation of the public except for health emergency or unavoidable work needs [20]. Shopping centers, educational institutions, and sporting events were temporarily closed to reduce the human-to-human transmissions and citizens were asked to stay at home.

The response strategies of the social distancing and lock-downs implemented in Italy did not yield similar results as those in China or Korea [21]. The study of Reis et al. [21] estimated that the underreporting of cases was very high in Italy through the estimation of mathematical models. The insufficient support and cooperation from the public for the order of quarantine may have provided a negative impact on the transmission of COVID-19.

### 4.4. Type IV: Low Mitigation Effectiveness and High Severity

The COVID-19 transmission in the USA and Brazil was characterized with low $E_{o}$ including a long time to reach the peak, low drop rate of daily new cases, and high $S_{o}$. The USA seemed to lose time in the fight against COVID-19 transmission at the early stage. It took the USA 97 days to reach the peak of new confirmed cases. The long time to reach the peak may be attributed to the low number of viral tests performed at the early stage. Ferrier and Hwang [22] indicated that many states in the USA had insufficient testing levels and lacked COVID-19 testing kits at the end of April 2020. Due to the barrier of testing capacity, the pandemic severity was underestimated in the early stages of the pandemic. The situation was gradually improved in March 2020 and tests per day increased roughly 10-fold between 15 March and 31 March 2020 [23]. Before 28 February 2020, no testing policy was employed. During a short period from 28 February to 4 March 2020, the testing policy only allowed those who both (a) had symptoms and (b) met specific conditions (e.g., contacts with a confirmed case, returned from overseas) to get viral tests. After 4 March 2020, open public testing was employed [6] and then the daily new confirmed cases reached a peak on 26 April 2020. However, the drop rate is not obvious after the peak. The maximum drop rate was only 48.44%. The fluctuation of daily new confirmed cases after the peak in the USA implies that a vicious cycle was happening.

The possible cause for the low effectiveness at the latter stage and the high pandemic severity may be attributed to the less effective contacts tracing systems [13]. Moreover, the response strategies among the states and between the state and the federal governments are and various without coordination. In response to the rising emergency, some states announced the order to stay-at-home to prevent the transmission of the infection, while others did not. Many people did not cooperate with the order to stay at home or to practice social distancing.

The major cause for the low $E_{o}$ and high $S_{o}$ in Brazil may be attributed to less vertical integration between the federal and state governments. The president of Brazil believes in natural immunity to COVID-19 and strongly suggests Brazilians to practice it. He prompted an advertising campaign against social distancing measures to fight against COVID-19 [24]. Thus, the implementation of social distancing and lockdown of cities implemented in Brazil reduced contacts only by 40%, while a reduction of contacts in Italy and Korea were estimated to be 75% and 90%, respectively [21]. Public health experts call for effective response strategies like lockdown of cities, but many states have not imposed the order to stay at home due to the opposition from the president [25]. In fact, the growing number of new confirmed cases currently occuring in Brazil implies that the anti-pandemic strategies of natural immunity completely fails at defeating the COVID-19.

The high mitigation effectiveness performed by Korea and China implies that the response strategies of massively extensive COVID-19 tests with effective contacts tracing system may play a key role in mitigating the transmission of COVID-19. Massive and extensive testing for COVID-19 may identify hotspots of infection in the country and thus appropriate response strategies may be implemented. Insufficient COVID-19 tests performed at the early stage may distort the real state of the pandemic and bring about underestimated infection rates. The asymptomatic infected who lurk in public places may contact more people and yield more confirmed cases a short time later. Eventually, it takes a longer time to reach the peak of the daily new cases and much more time to decline from the peak.

Furthermore, the non-pharmaceutical interventions such as lockdown, staying at home, social distancing, school closures, etc. also may prevent the transmission of the disease and cut down the path of infection. This paper confirms that the strict implementation of the non-pharmaceutical interventions provides substantial effectiveness in mitigating the transmission of COVID-19. The conclusion in this paper coincides with those of previous studies (e.g., [26,27]). Cooperation from the public is vital to a successful lockdown as the lockdown or staying at home may incur economic costs and damage the economy. Thus, public awareness of the pandemic severity and the necessity of the response strategies adopted by the governments are also important to mitigate the COVID-19 transmission.

## 5. Conclusions

Many countries have contributed significant efforts to developing a vaccine or viable treatment options for COVID-19, but no vaccine or explicit medicines for the treatment of COVID-19 are available [28,29,30,31]. In the future, the role of vaccination in mitigating the COVID transmission may be an area of research. Before vaccine and pharmaceutical treatments for COVID-19 are successfully developed, the outcome of death is inevitable. Thus, the response strategy of using non-pharmaceutical interventions is a necessity to reduce COVID-19 transmission and the negative impact on medical systems [10,32,33].

During the development of the pandemic, citizens with a low socio-economic status are more susceptible to contracting the disease of COVID-19 and have a higher probability of dying due to an insufficient supply of medical services. Medical and socio-economic inequality may have multiple effects on the spread and lethality of COVID-19. Currently, data of individual characteristics such as age, gender, race, inhabitant conditions, and medical history are not accessible. In the future, this research may be extended to focus on the effect of demographical factors and medical systems on the COVID-19 infection and mortality. Previous studies found that the transmission of SARS and MERS was highly association with air quality [34], and thus the impact of environmental conditions on COVID-19 may be an area of focus in the future. Only six sample countries are analyzed and compared in this paper. If the response strategies are provided in other countries, a more broad study may be conducted in the future.

## Figures and Tables

**Table 1:** The date of the first confirmed case in the six countries.

Country	Date of the First Confirmed Case
China	8 December 2019 (estimated)
Japan	15 January 2020
Korea	20 January 2020
Italy	31 January 2020
USA	23 January 2020
Brazil	27 February 2020

**Table 2:** The mitigation effectiveness for the reduction of the transmission after the peak (unit: days).

	China	Korea	Japan	Italy	USA	Brazil
$E_{e}$ (days)	60	40	87	52	97	129
Date of the peak	02/05	02/29	04/12	03/22	04/26	07/04
Days for the drop of the pandemic by 50%	9 (69th)	9 (49th)	15 (102th)	24 (76th)	Not yet	Not yet
Days for the drop of the pandemic by 80%	16 (76th)	13 (53th)	25 (112th)	51 (103th)	Not yet	Not yet
Days for the drop of the pandemic by 90%	27 (87th)	39 (79th)	36 (123rd)	63 (115th)	Not yet	Not yet
Days for the drop of the pandemic by 95%	27 (87th)	42 (82th)	42 (129th)	72 (124th)	Not yet	Not yet
$E_{f}$	99.37% (96th)	99.55% (107th)	96.44% (129th)	95.87% (130th)	48.44% (109th)	38.55% (132nd)
$E_{o}$	High	High	Middle	High	Low	Low

Note: The parentheses represent the days since the first confirmed case for each country.

**Table 3:** The pandemic severity in each country (unit: case per 10^6^ persons per day).

	China	Korea	Japan	Italy	USA	Brazil
$S_{e}$	0.28	1.54	0.61	17.04	28.01	54.59
$S_{f}$	0.57	1.75	1.71	37.20	77.54	148.69
$S_{o}$	0.32	1.61	0.92	30.23	40.50	74.87
*d*	0.02	0.04	0.05	4.24	2.41	2.32
Categorization of $S_{o}$	Low	Low	Low	High	High	High

**Table 4:** The characteristics of the pandemic in the selected countries.

	China	Korea	Japan	Italy	USA	Brazil
Type	I	I	II	III	IV	IV
Mitigation effectiveness	High	High	Middle	High	Low	Low
Pandemic severity	Low	Low	Low	High	High	High
